# Enhanced YOLOv5 Object Detection Algorithm for Accurate Detection of Adult *Rhynchophorus ferrugineus*

**DOI:** 10.3390/insects14080698

**Published:** 2023-08-09

**Authors:** Shuai Wu, Jianping Wang, Li Liu, Danyang Chen, Huimin Lu, Chao Xu, Rui Hao, Zhao Li, Qingxuan Wang

**Affiliations:** 1 School of Computer and Communication Engineering, University of Science and Technology Beijing, Beijing 100083, China; 2 Hainan Key Laboratory of Tropical Oil Crops Biology, Coconut Research Institute of Chinese Academy of Tropical Agricultural Sciences, Wenchang 571339, China; 3 Shunde Innovation School, University of Science and Technology Beijing, Foshan 528399, China

**Keywords:** red palm weevil, YOLOv5, attention mechanism, detection

## Abstract

**Simple Summary:**

The red palm weevil is an exotic and highly endangered pest that is extremely harmful to palm plants. In order to effectively control this pest, we propose an algorithm to automatically detect and count adult red palm weevils in traps. Previously, the trapping and counting of adult red palm weevils was done manually. The population density and damage level were then inferred from the number of adults trapped to guide control efforts. However, the efficiency of this method is very low. The algorithm proposed in this paper solves the drawbacks of manual counting, and the recognition accuracy reaches 93.8%, which also improves the efficiency of agricultural monitoring.

**Abstract:**

The red palm weevil (RPW, *Rhynchophorus ferrugineus*) is an invasive and highly destructive pest that poses a serious threat to palm plants. To improve the efficiency of adult RPWs’ management, an enhanced YOLOv5 object detection algorithm based on an attention mechanism is proposed in this paper. Firstly, the detection capabilities for small targets are enhanced by adding a convolutional layer to the backbone network of YOLOv5 and forming a quadruple down-sampling layer by splicing and down-sampling the convolutional layers. Secondly, the Squeeze-and-Excitation (SE) attention mechanism and Convolutional Block Attention Module (CBAM) attention mechanism are inserted directly before the SPPF structure to improve the feature extraction capability of the model for targets. Then, 2600 images of RPWs in different scenes and forms are collected and organized for data support. These images are divided into a training set, validation set and test set following a ratio of 7:2:1. Finally, an experiment is conducted, demonstrating that the enhanced YOLOv5 algorithm achieves an average precision of 90.1% (mAP@0.5) and a precision of 93.8% (P), which is a significant improvement compared with related models. In conclusion, the enhanced model brings a higher detection accuracy and real-time performance to the RPW-controlled pest pre-detection system, which helps us to take timely preventive and control measures to avoid serious pest infestation. It also provides scalability for other pest pre-detection systems; with the corresponding dataset and training, the algorithm can be adapted to the detection tasks of other pests, which in turn brings a wider range of applications in the field of monitoring and control of agricultural pests.

## 1. Introduction

The Red Palm Weevil (RPW, *Rhynchophorus ferrugineus*) is an internationally recognized and highly hazardous quarantine pest that targets palm trees (Arecaceae). The RPW is widely distributed and has infested several countries. In China, especially in Hainan and other regions, extensively cultivated palm plants with an annual production value in the billions of dollars face immeasurable potential economic losses from a widespread infestation. It is known for its extensive distribution and destructive burrowing behavior, making its control extremely challenging [1]. As a result, scientists at home and abroad have conducted numerous studies on the RPW. For example, the distribution and bioecology of the RPW are characterized in [2], where it is shown that the RPW and its ability to spread easily, with a theoretical uninterrupted flight distance of up to 1.5 km, leads to an even greater destructive potential. In addition, the challenges posed by the RPW necessitate the implementation of more comprehensive control measures to mitigate its impact on major palms worldwide [3]. Therefore, in order to effectively control the RPW, some studies on common monitoring methods were analyzed in [4]. The general methods for monitoring larvae inside the tree are physical detection, such as CT scanning, and radar. On the other hand, the most common and effective method for monitoring adults outside the tree is pheromone trap technology, which is discussed in [5,6,7].

However, relying solely on trapping techniques is not sufficient to prevent the continuous spread of this pest in a timely manner. To address this issue, scientists have found that the period when the adults are briefly exposed to the tree is a critical time for monitoring and control. Adults can be monitored in order to grasp the population density and take appropriate measures. As for the monitoring method of the early stages of the adult RPW, pheromone trap monitoring technology is more mature both at home and abroad. This technique utilizes pheromones to specifically attract adult RPWs to the trap. According to the number of trapped adults, the population density and damage level within the monitoring area can be inferred to guide the control. However, counting the number of adults in the traps set up for the purpose of monitoring the population density of adults mostly relies on manual counting on a regular basis, which is a less efficient and intelligent method. A method of automatic detection and counting is required to improve efficiency.

Currently, the common automatic counting systems that can be integrated into field insect trapping devices include several approaches. Firstly, there are counting systems based on traditional image processing algorithms. However, these methods exhibit limited effectiveness in complex field scenarios with significant target variations, leading to false positives or missed detections [8]. Secondly, there are sensor-based counting systems, but they lack sufficient precision in counting densely distributed or small targets. It is evident that traditional field-deployed counting systems still have certain drawbacks, making it challenging to detect and count the RPW accurately in different scenarios [9].

Deep learning-based target detection techniques have been widely used in the field of plant pest and disease identification. By using target detection algorithms, automated pest detection can be realized, and detection efficiency can be improved. Target detection techniques can quickly process large amounts of image data and accurately identify targets, thus avoiding time-consuming and costly manual detection. Target detection algorithms can be broadly categorized into two types: the first type is the two-stage target detection algorithm represented by R-CNN (Region-CNN) [10], which includes Fast R-CNN [11], Faster R-CNN [12], and other similar models. These algorithms generate candidate frames and then execute convolutional networks to identify the detected objects. Due to the high computational complexity of this type of algorithm, it is not suitable for real-time detection. The second type is the one-stage target detection algorithm represented by the SSD (Single Shot MultiBox Detector) [13] and YOLO (You Only Look Once) [14,15,16,17] series. This type of algorithm directly generates the class probability and position information of the object, which can directly obtain the final detection result after a single detection. Compared to the two-stage algorithm, this type of algorithm is faster, but there may be a loss of accuracy. Among the first stage detection algorithms, the YOLO family serves as a representative framework for single-stage detection, which is faster and more stable compared to SSD neural networks [18]. YOLO is a high-performance general-purpose target detection model. YOLOv1 [14] uses a single-stage detection algorithm to accomplish the two tasks of localizing a target and classifying target objects. Subsequently, YOLOv2 [15] improved the algorithm in three aspects: more accurate prediction, faster speed, and more targets identified compared to YOLOv1. YOLOv3 [16] accelerated the implementation of object detection by introducing multi-scale prediction, core network optimization, and loss function improvement. YOLOv4 [17] presented an efficient and fast object detection model that significantly reduced the computational number of parameters, making it easier to deploy on general-purpose and hardware devices. Compared to YOLOv4, YOLOv5 has a smaller and more flexible architecture, faster image inference, and is closer to the natural production life. In addition to this, it has been widely applied due to its advantages, such as fast average detection, flexibility, and rapid deployment [19]. In recent years, YOLOv5 has been commonly used in research on pest and disease detection. For example, a target detection system based on YOLOv5 for detecting crop hazardous pests and their classification was proposed in [20]. A YOLOv5 model with 100% detection accuracy was created for detecting rice foliar pests in [21]. In addition, a novel pest detection method based on improved YOLOv5 was analyzed in [22], which achieves high accuracy pest detection, among others. It is shown that the method can be applied to the rapid real-time detection of RPW in complex natural environments due to the high real-time nature of the YOLOv5 target detection model, which facilitates the efficiency of monitoring equipment.

However, the actual environment in which RPW lives is complex. The main problems are: (1) small target objects, with the whole target taking up a smaller proportion of the whole image; (2) severe object occlusion, with individuals obscuring and overlapping each other; and (3) complex backgrounds, increasing the difficulty of feature target extraction [17]. At the same time, considering YOLOv5’s fast speed, which leads to some loss of accuracy, it performs poorly on the information of small targets and suffers from poor accuracy and low recall in identifying object locations [18]. Therefore, the addition of a quadruple down-sampling layer to the backbone network of YOLOv5 is proposed to improve the semantic information of small targets and, thus, make the prediction of the model more accurate. In addition to this, YOLOv5 suffers from insufficient bounding box localization as well, and has difficulty distinguishing between overlapping detection objects, especially objects such as insects that are heavily occluded [23]. However, the presence of an attention mechanism can effectively solve these problems. When processing information, the attention module resembles the human visual attention mechanism by scanning the global image to obtain the target area that needs to be focused on and then devoting more attention resources to this area to obtain more detailed information related to the target while filtering out the secondary data to improve the model’s effect. With the development of machine learning, the combination of attention mechanism and deep learning has become more and more extensive, and adding the attention mechanism to a model can be used as a means to improve performance. Accordingly, Squeeze and Excitation Net (SE Net) [24] and Convolutional Block Attention Module (CBAM) [25] were integrated into the convolutional module of YOLOv5 to implement the learning of target features and location features in the channel dimension and global spatial dimension, respectively. By adding a down-sampling layer combined with a feature fusion network and adding an attention mechanism for multi-dimensional feature learning, the problem of difficult feature extraction due to occlusion and complex backgrounds is skillfully solved, ultimately improving the detection performance.

In order to detect and control the RPW more accurately and efficiently, an enhanced YOLOv5 target detection algorithm is proposed in this paper. Firstly, the dataset of RPW is collected. At the same time, the dataset is subjected to different levels of luminance conversion to make the pest target detection model independent from the light diversity of the field environment. Then, the contrast of the RPW’s image is increased by different magnitudes to better represent the clarity, gray scale, and texture details. Additionally, the images are randomly rotated at multiple angles to enrich the multiple morphologies of the insect. Multiple data enhancement methods are used to greatly enrich the number of samples for RPW recognition in complex backgrounds. Next, an enhanced YOLOv5 neural network model was constructed in PyTorch. The main improvements are (1) enriching the semantic information of small targets by adding quadruple down-sampling layers and improving the feature pyramid structure to improve the model’s detection ability for small targets, and (2) introducing the attention mechanism to enhance the feature extraction ability of the model. Then, the dataset is divided into a training set and test set in the ratio of 7:1. The enhanced model is subjected to comparative experiments, and the experimental results are evaluated using commonly used machine learning algorithm evaluation metrics, such as Precision (P), Recall (R), and mAP. Finally, the evaluation results show that the enhanced algorithm has the highest detection accuracy and enhances the real-time detection of RPW in complex environments.

The remainder of the paper is organized as follows: Section 2 describes the structure of YOLOv5 and the rationale for improving the content. Section 3 describes the dataset acquisition and enhancement process as well as the experimental procedure. Section 4 presents the experimental results and discussion. Section 5 summarizes the paper.

## 2. Materials and Methods

### 2.1. YOLOv5

**Figure 1:**
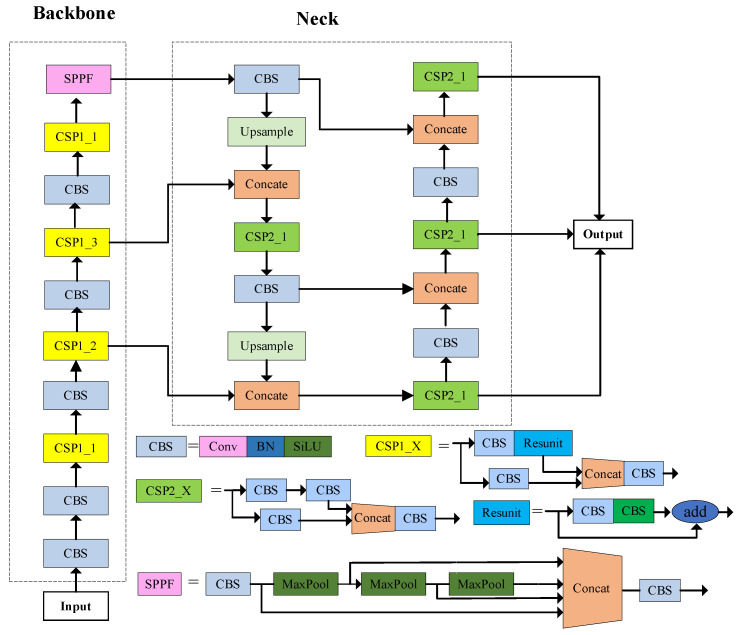
YOLOv5s network structure.

YOLOv5 is a one-stage target detection algorithm with a network structure consisting of inputs, trunks, necks, and outputs. It includes four network models: YOLOv5s, YOLOv5m, YOLOv5l, and YOLOv5x, listed in order of increasing network depth and weight file size [20]. To realize high performance on real-time detection, we chose the YOLOv5s model for experimental training from the perspective of minimizing computational cost and network weighting in this paper. The network structure of the YOLOv5s model is depicted in Figure 1.

#### 2.1.1. Input

The input part of the network structure in Figure 1 contains an image preprocessing stage that scales the input image to the input size of the network and performs operations, such as normalization, including mosaic data enhancement operations, adaptive anchor frame calculation, and adaptive image scaling methods [18]. Mosaic data enhancement increases the complexity of the data by combining and arranging our images. In addition, an adaptive anchor frame calculation is used to derive the best anchor frame values best suited for different training sets and the adaptive image scaling is used to automatically fill the images with black borders, scale them uniformly to the standard size, and finally feed them into the detection network.

#### 2.1.2. Backbone

The backbone network part in Figure 1 consists of CSPDarknet53, which is responsible for extracting features from target objects [26]. It mainly consists of the CBS module and the SPPF (Spatial Pyramid Pooling Fast) module. The CBS module includes the Conv2d module, BN (Batch Normalization) layer, and SiLU activation function [27]. SPPF is a modified version of the SPP (Spatial Pyramid Pooling) structure, in which the input features are passed through a series of maximum pooling layers, and the input as well as the output feature layer sizes are spliced and fused in the channel direction. SPPF differs from SPP in that its output after each pooling becomes the input of the next pooling, and then they are stitched and fused together. This modified structure allows SPPF to address the target multi-scale problem to some extent, while also being faster than SPP.

#### 2.1.3. Neck Network

**Figure 2:**
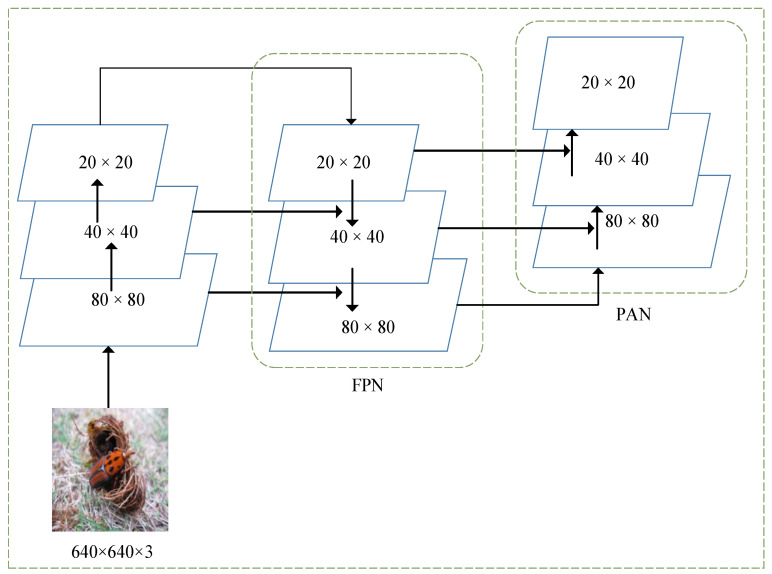
PAN and FPN structure.

In Figure 1, the neck part mainly consists of Feature Pyramid Network (FPN) and Path Aggregation Network (PAN), which is the feature fusion network of the model. In convolutional neural networks, different convolutional layers yield feature maps with distinct target features. Shallow convolutions produce feature maps with high resolution and relatively rich positional information, but less prominent semantic information. Deep convolutions, on the other hand, generate feature maps with lower resolution but rich semantic information, at the cost of losing significant positional details. Consequently, shallow convolutional layers are capable of distinguishing simple objects, while deep convolutional layers excel in discerning complex objects. The fusion of information between shallow and deep convolutional layers is advantageous for object detection, which is the principle of feature fusion networks [23]. As shown in Figure 2, the FPN transfers strong semantic features from top to bottom and the PAN conveys the strong positioning features of the target from bottom to top. By fusing top-down and bottom-up feature information, the model can learn features better and improve the accuracy of the model for small target detection.

#### 2.1.4. Output

In Figure 1, The output part is responsible for generating the detection results for the target objects. It employs the Generalized Intersection over Union (GIoU) loss function to compute the bounding box loss. In addition, the Non-Maximum Suppression (NMS) operation is used to eliminate duplicate detections and achieve the final output detection results.

### 2.2. The Improved Network Model

#### 2.2.1. Introduce the Quadruple Down-Sampling Layer

**Figure 3:**
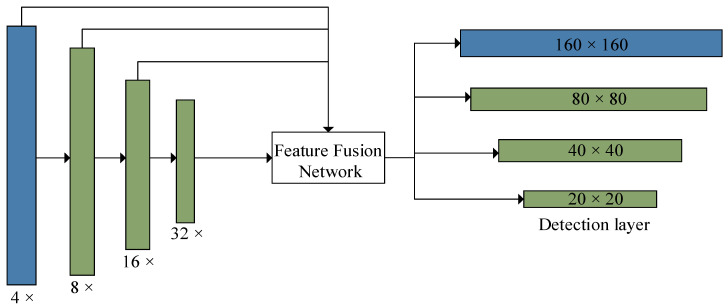
The improved backbone network.

The backbone network of YOLOv5 focuses on multi-scale prediction of the input images, where images of different scales are fed to the input and down-sampled by factors of 8, 16, and 32 to obtain feature images of three different scales, which are then fed to a feature fusion network for target recognition. The process of feature fusion is depicted in Figure 2. It is known within the idea of a feature pyramid network [28] that the feature pictures obtained after multiple convolutions contain rich semantic information, however, due to the process of down-sampling, some target location information may be lost, which makes it difficult to detect small target objects successfully [23]. This issue is particularly relevant in complex field environments where targets like RPW are too small, so to address this challenge, the addition of a quadruple down-sampling layer to the backbone network of YOLOv5 is presented, which can enhance the detection capability of small target features, and the network structure is illustrated in Figure 3. By adding a quadruple down-sampling layer, the original image is fed into a feature fusion network to obtain a feature map in a new dimension. The feature map has a small perceptual domain and relatively rich position information, which improves the detection of small targets [29].

#### 2.2.2. Introduce the Squeeze-and-Excitation Net

**Figure 4:**
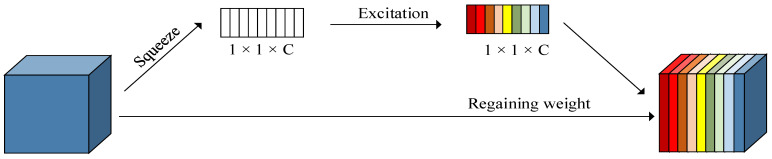
Squeeze-and-Excitation structure.

Based on the YOLOv5 framework, the Squeeze-and-Excitation Net (SE Net) is introduced into the backbone network. SE Net belongs to channel attention [24], whose process mainly consists of two parts: squeezing and stimulation, as illustrated in Figure 4. Firstly, the input image features are compressed, followed by feature learning of the compressed feature map to obtain learning weights, and finally the original feature map is multiplied by the learned weights to obtain the final features. This technique allows the model to prioritize the most informative and distinctive features while ignoring less relevant and secondary features. The complex field environment and the presence of other insects can potentially interfere with RPW’s feature detection abilities, while the SE channel attention module can enhance the feature extraction capability of the current task.

#### 2.2.3. Introduce the Convolutional Attention Module

**Figure 5:**
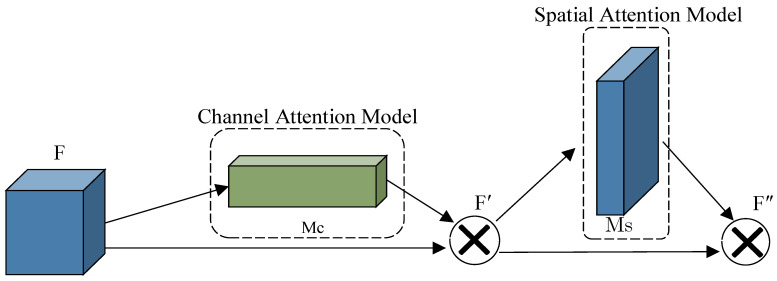
Structure illustration of the channel and spatial attention module.

Based on the YOLOv5 framework, the Convolutional Block Attention Module (CBAM) is introduced into the backbone network as well. CBAM consists of channel attention and spatial attention modules, as shown in Figure 5, and this structure can better extract the weight distribution in feature learning and improve the feature extraction ability of the model for small target samples [25]. As can be seen from Figure 5, for the input feature F, firstly, the average pooling and maximum pooling operations are performed by the channel, and the one-dimensional channel attention M_c_ is obtained after aggregating the spatial information of the feature map. Secondly, M_c_ is multiplied with the input elements to obtain the adjusted feature map F′, and then the pooling operation is performed on F′ by space to obtain two two-dimensional vectors, stitching them together and performing a convolution operation to generate two-dimensional space note M_s_. Finally, M_s_ is multiplied with F′ by element to obtain the fused feature F″. The CBAM process of generating attention can be described as:

(1) $$F\prime =M_{c}(F)⊗F$$

(2) $$F\prime \prime =M_{s}(F\prime )⊗F\prime$$

where ⊗ denotes the corresponding element multiplication.

The large number and variety of insects in the field often result in stacks. However, since the RPW is tiny in size and hidden from the target, making it difficult to detect after generating individual stacking. Therefore, the CBAM module can enhance the feature expression of the obscured RPW and improve the recognition performance of target samples.

### 2.3. An Improved RPW Detection Model Based on YOLOv5

**Figure 6:**
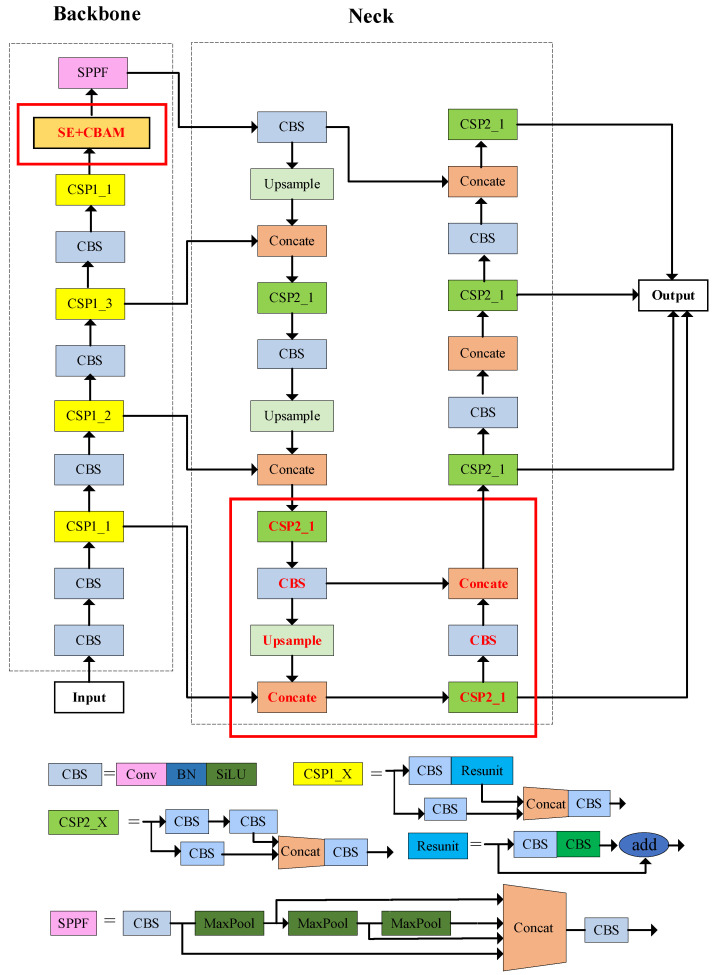
Structure of the improved YOLOv5s network model. The improvements are shown in the red box.

Finally, the overall network structure is enhanced based on the YOLOv5s network, as illustrated in Figure 6. The red boxes in the figure indicate the improved parts. This network improves the detection of small targets by adding a quadruple down-sampling layer to the backbone network, and also extracts useful location information with the introduction of the SE and CBAM attention modules. Through the above improvements, the overall detection performance is enhanced.

## 3. Experiment

### 3.1. Data Acquisition and Enhancement

**Figure 7:**
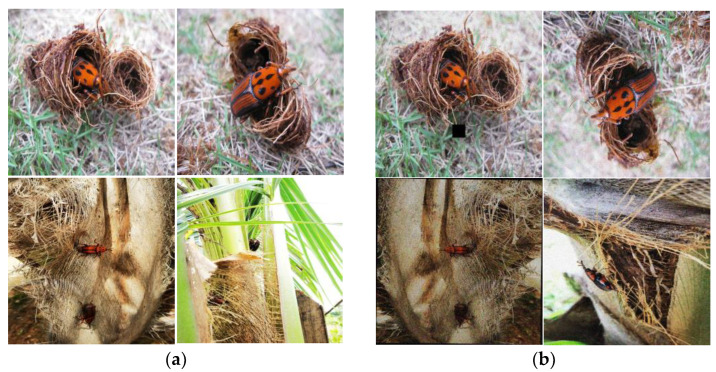
Data processing results: (**a**) partial data set (**b**) selected data sets for data enhancement.

The dataset is collected manually using the rear camera of a phone, with an image resolution of 3024 pixels × 3024 pixels. To ensure compatibility with different deep learning frameworks, the images are uniformly processed to a size of 640 pixels × 640 pixels, such that images of different sizes are converted to the same size to create a consistent training dataset. The final dataset consists of 305 images of the RPW, taken from different angles and in different scenes, and the examples of images from the dataset are shown in Figure 7a. However, the current dataset cannot meet the demand of practical detection; to improve the model training, the dataset needs to be enriched. The common means of data enhancement are: (1) flipping: randomly flipping the images (0–180°); (2) adding noise: adding noise to the original image; common noises are pretzel noise, Gaussian noise, etc; (3) rotating: randomly rotating the picture from 0–360°; (4) scaling: changing the size of the image according to the proportions; and (5) brightness and contrast changes: adjusting brightness, contrast, etc. [18]. Finally, 2600 images of RPW samples are obtained after data expansion, after which, these images are divided into a training set, validation set, and test set in the ratio of 7:2:1, where the training set has 1520 images, the validation set includes 520 images, and the test set includes 260 images [30], and some of the images are shown in Figure 7b.

### 3.2. Evaluation Indicators

To ensure the accuracy of the experimental results, this paper employs several metrics to evaluate the training outcomes of the RPW experiment. The commonly used evaluation metrics include Intersection over Union (IoU), Precision (P), Recall (R), and mean Average Precision (mAP), which can be calculated as follows:

(3) $$P=\frac{TP}{TP+FP}$$

(4) $$R=\frac{TP}{TP+FN}$$

(5) $$AP=\int_{0}^{1}P(R)dR$$

(6) $$mAP=\frac{\sum_{i=1}^{N}AP_{i}}{N}$$

(7) $$IoU=\frac{B_{a}\cap B_{b}}{B_{a}\cup B_{b}}$$

where TP denotes the number of positive samples that are correctly detected as positive, FP denotes the number of negative samples that are mistakenly detected as positive, FN denotes the number of positive samples that are missed and wrongly detected as negative. AP value refers to the area of the P-R curve, and in Equation (6), the value of mAP is obtained by averaging all categories of AP, and N represents the total number of types detected. The larger the value of mAP in this experiment, the better the algorithm detected and the higher the recognition accuracy. B_a_ denotes the area of the predicted frame, while B_b_ denotes the area of the ground truth frame. The IoU ratio indicates the degree of overlap between the predicted and ground truth frames. A higher IoU value suggests greater accuracy of the prediction. The mAP at an IoU threshold of 0.5 (mAP@0.5) signifies that non-maximum suppression (NMS) is applied with an IoU threshold greater than or equal to 0.5, and mAP@0.5:0.95 indicates that the IoU threshold was varied from 0.5 to 0.95 in increments of 0.05, and the resulting average value is computed [31].

### 3.3. Experimental Implementations and Settings

In this paper, a small-target detection layer is added to the network, while the SE Net and CBAM modules are also incorporated into the backbone architecture of YOLOv5s. Furthermore, comparative experiments are conducted with the original YOLOv5s, all of which are performed using the PYTORCH deep learning framework [32]. The model is trained on a hardware platform consisting of an NVIDIA GeForce GTX 1070 graphics card, and the operating system used for training is Windows 10. To ensure experimental rigor, consistent parameter settings are used for all ablation experiments, and experimental platforms and the model training parameters are set as follows: learning rate = 0.01, momentum = 0.937, weight decay = 0.0005, batch size = 8, and number of iterations = 1000.

## 4. Experimental Results and Discussion

The detection performance of the improved model is compared with the original model, and the RPW dataset is used to evaluate the performance of the above models with the following evaluation metrics: Precision, mAP@0.5 and mAP@0.5:0.95.

Table 1 summarizes the various models and their corresponding descriptions as well as comparative results of the ablation experiments. Among them, YOLOv5s-4x represents the YOLOv5s model with an additional four-fold down-sampling layer. YOLOv5s-4x-SE indicates the inclusion of both a four-fold down-sampling layer and SE attention mechanism. YOLOv5s-4x-CBAM includes a four-fold down-sampling layer and CBAM attention mechanism. Lastly, YOLOv5s-4x-SE-CBAM signifies the model with all three components: a four-fold down-sampling layer along with SE and CBAM attention mechanisms. Through comprehensive analysis, the ablation experiments in Table 1 show that the detection accuracy P tends to increase with the improvement of the algorithm. Compared to the original YOLOv5 algorithm, adding a small-target detection layer can improve the accuracy and average precision of detection, so the subsequent experiments are improved on this basis. In addition, the addition of the attention mechanism can also improve the detection accuracy. From the results, it can be seen that the two attention mechanisms are comparable in improving the detection accuracy. However, it is worth noting that although the CBAM network has a higher accuracy than the SE network, it does not improve the average accuracy of mAP@0.5. Nevertheless, the algorithm proposed in this paper greatly improves the detection accuracy compared to the original network, and the precision improves by 2.5% and the mAP@0.5 improves by 1.3%. This gives the present algorithm a significant advantage over the unimproved YOLOv5s.

**Figure 8:**
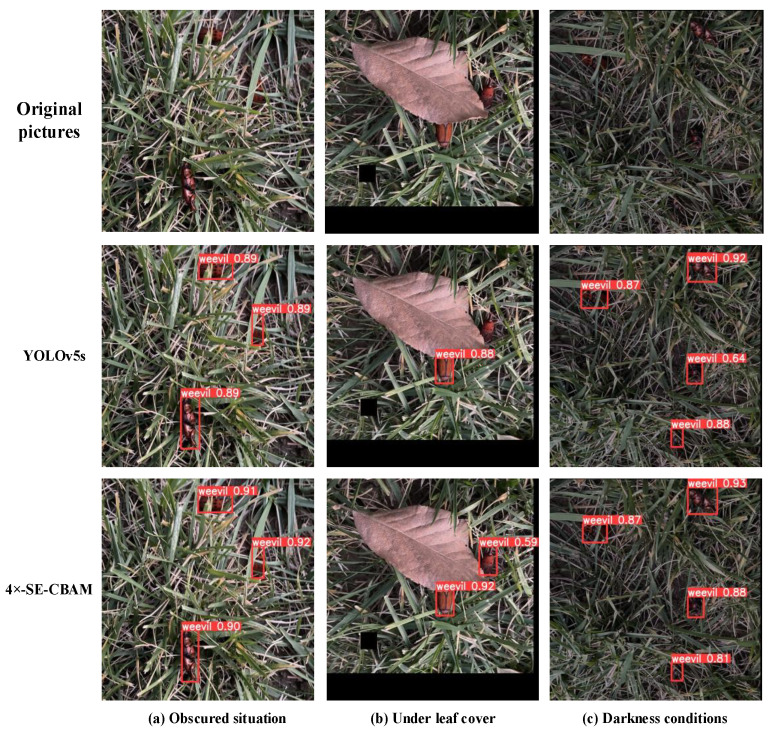
Detection results. The red frame in the graph shows the percentage of detections.

In complex natural environments, a variety of insects are mixed together and the phenomenon of multiple insect stacking is produced. This leads to the obscuring of the physical signs of a single individual, which causes the individual features to become less obvious. As a result, the detection network cannot accurately detect the features, causing the detection accuracy of the model to decrease. The algorithm proposed in this paper, on the other hand, adds a quadruple down-sampling layer to the original YOLOv5s algorithm, which is combined with a feature fusion network to make the features of small targets easier to extract. At the same time, the SE, and CBAM attention mechanisms are added to extract features from the multi-channel dimension, which solves the problem of difficult extraction of features caused by the individual stacking occlusion problem. Compared to the original YOLOv5s network, the detection accuracy of the model is improved. To evaluate the effectiveness of the proposed model in this challenging scenario, three representative RPW images from the test set are selected, which include a variety of realistic scenarios that are difficult to detect. These three images are, in order, a field environment with a complex background and occluded insects, insects with localized features occluded by leaves, and an image that is poorly lit at night, resulting in features that are not obvious. Then these three sets of images under the original algorithm and the improved algorithm in this paper are tested, and the detection results of the two models are shown in Figure 8 [33]. As a consequence, our data analysis and detection results demonstrate that the proposed algorithm in this paper achieves significantly higher detection accuracy compared to the YOLOv5s network. Specifically, in the case of mixed and obscured environments, as can be seen from Figure 8a, the accuracy of the original YOLOv5s network reaches up to 89%, while the proposed algorithm achieves an accuracy of 92%, which is a 3% improvement in the actual detection accuracy compared to the original algorithm. Furthermore, our findings indicate that the original model fails to detect the obscured RPW, whereas the improved algorithm successfully identifies the sample in Figure 8b. Notably, our nightly detection accuracy reaches 93% in Figure 8c, which is a 1% improvement compared to the original model’s detection accuracy. In summary, the improved model outperforms the original model in terms of detection effectiveness.

## 5. Conclusions

Given the challenges associated with early-stage detection and control of the RPW, this paper proposes an improved algorithm for YOLOv5. The YOLOv5s model is adopted as the basic framework, and the detection capability of small targets is improved by adding a quadruple down-sampling layer to the backbone network; an attention mechanism-based feature extraction module is designed, and the SE and CBAM attention mechanism are added to improve the feature extraction capability of the model. Through the validation experiments of the sample data on different models, the detection accuracy P of the proposed algorithm reaches 93.8% and the average accuracy mAP@0.5 reaches 90.1% on the dataset, and mAP@0.5 and mAP@0.5:0.95 are improved by 1.4% and 0.8%, respectively, compared with the original network. The experimental results demonstrate that the proposed algorithm achieves high accuracy in detection and can effectively support field monitoring efforts. These findings underscore the potential value of the proposed algorithm in real-world agricultural applications.

In addition, the proposed algorithm has a wider application prospect in terms of practicality and generalization. Currently, in regions like Pakistan, pesticide usage is the primary method for managing RPWs, whereas in South America and Brazil, pheromone-based trapping is the main approach. However, the RPW trapping and detection system proposed in this study can be applicable to areas where host plants are distributed in remote locations, making manual counting inconvenient. It can also be utilized in regions with extensive and expansive monitoring areas, resulting in low efficiency in manual surveillance. In the meantime, to enhance the applicability of the algorithm in other pest management systems, we will extend the dataset to cover more species and targets. Given the high morphological similarity between this pest and closely related species, current models may face challenges in accurately categorizing and detecting these insects. Therefore, strategies for integrating additional modal information, such as images, will need to be further investigated to enhance the accuracy of insect classification and detection. This involves collecting supplementary data from various modalities and conducting preprocessing and feature fusion to enable the model to learn richer representations from multiple sources. Ultimately, we will use deep learning models to train and optimize the fused data to improve the performance of the algorithm for morphologically similar species recognition.

## Figures and Tables

**Table 1:** Results of ablation experiments.

Model	Precision	Recall	mAP@0.5	mAP@0.5:0.95
YOLOv5s	0.913	0.828	0.888	0.485
YOLOv5s-4x	0.923	0.813	0.893	0.486
YOLOv5s-4x-SE	0.928	0.811	0.878	0.454
YOLOv5s-4x-CBAM	0.932	0.795	0.849	0.437
YOLOv5s-4x-SE-CBAM	0.938	0.834	0.901	0.489

## Data Availability

Relevant data are available from the authors upon reasonable request.
